# Fucoidan Improves D-Galactose-Induced Cognitive Dysfunction by Promoting Mitochondrial Biogenesis and Maintaining Gut Microbiome Homeostasis

**DOI:** 10.3390/nu16101512

**Published:** 2024-05-17

**Authors:** Yan Xu, Meilan Xue, Jing Li, Yiqing Ma, Yutong Wang, Huaqi Zhang, Hui Liang

**Affiliations:** 1 School of Public Health, Qingdao University, Qingdao 266071, China; xy2514580989@163.com (Y.X.); lijing_95123@163.com (J.L.); mayiqing136@163.com (Y.M.); wyt687199@126.com (Y.W.); huaqi_erin@163.com (H.Z.); 2 Basic Medical College, Qingdao University, Qingdao 266071, China; snowml@126.com

**Keywords:** D-galactose, fucoidan, cognitive impairment, mitochondrial biogenesis, gut microbiome

## Abstract

Recent studies have indicated that fucoidan has the potential to improve cognitive impairment. The objective of this study was to demonstrate the protective effect and possible mechanisms of fucoidan in D-galactose (D-gal)-induced cognitive dysfunction. Sprague Dawley rats were injected with D-galactose (200 mg/kg, sc) and administrated with fucoidan (100 mg/kg or 200 mg/kg, ig) for 8 weeks. Our results suggested that fucoidan significantly ameliorated cognitive impairment in D-gal-exposed rats and reversed histopathological changes in the hippocampus. Fucoidan reduced D-gal-induced oxidative stress, declined the inflammation level and improved mitochondrial dysfunction in hippocampal. Fucoidan promoted mitochondrial biogenesis by regulating the PGC-1α/NRF1/TFAM pathway, thereby improving D-gal-induced mitochondrial dysfunction. The regulation effect of fucoidan on PGC-1α is linked to the upstream protein of APN/AMPK/SIRT1. Additionally, the neuroprotective action of fucoidan could be related to maintaining intestinal flora homeostasis with up-regulation of *Bacteroidota*, *Muribaculaceae* and *Akkermansia* and down-regulation of *Firmicutes*. In summary, fucoidan may be a natural, promising candidate active ingredient for age-related cognitive impairment interventions.

## 1. Introduction

Aging is an essential factor in the gradual decline of brain function and is associated with dementia, progressive cognitive dysfunction and memory impairment [1,2]. Accumulating evidence shows that oxidative stress, inflammation and mitochondrial dysfunction are closely related to some age-related neuronal diseases [3,4]. The direct consequence of oxidative stress includes DNA oxidation, lipid peroxidation and protein oxidation, which leads to neuronal cell death [5,6]. Moreover, excessive reactive oxygen species (ROS) can activate dynamin-related protein 1 (DRP1), result in an increase in mitochondrial fragmentation and mitochondrial dynamic imbalance [7], and can damage mitochondrial DNA (mtDNA) and impair mitochondrial biogenesis [8], which ultimately causes mitochondrial dysfunction [9,10]. Mitochondrial dysfunction leads to more ROS, thus creating a vicious circle of increasing ROS and mitochondrial dysfunction [11], which exacerbates neurodegeneration. Additionally, a wide range of studies have indicated that the gut microbiota has been demonstrated to take an essential part in the pathogenesis of age-related brain dysfunction [12,13]. The disturbance of intestinal flora results in increased inflammatory levels and promotes oxidative stress. Therefore, effectively attenuating oxidative stress, ameliorating mitochondrial dysfunction, improving inflammation and regulating gut microbiota might be a potential neurotherapeutic approach to treat age-related neurodegenerative diseases.

Injection of D-galactose is known to be a brain-aging model that induces and accelerates aging. D-gal is commonly revealed in dairy products and sugar beets as a monosaccharide sugar, and it is also present in the human body. Nevertheless, high concentrations of D-gal are oxidized into hydrogen peroxide and galactitol, which can result in increased ROS and induce oxidative stress [14,15]. Multiple studies have found that D-gal also decreases some antioxidant enzyme activities while increasing malondialdehyde (MDA) content [16,17]. In addition, high doses of D-gal induce neuronal mitochondrial dysfunction, leading to neuronal degeneration and a consequent decline in cognitive ability. Also, D-gal induced aging can alter the gut microbiota, which is consistent with naturally aging organisms [15,18]. Thus, D-gal-exposed rodents are widely used as an animal model to simulate age-related cognitive dysfunction and to evaluate the intervention effects of medication against cognitive impairment [19,20,21].

Fucoidan, a potential natural product, is a sulfated polysaccharide mainly made of sulfate groups and L-fucose. Fucoidan has an extensive spectrum of biological and pharmacological activities, including anticancer, anticoagulant, antiviral and anti-inflammatory activities [22,23,24,25]. It is highly reliant on the molecular weight, and the content and location of the sulfate groups of fucoidan for its biological activity [26]. A study has found that fucoidan can ameliorate the memory loss and learning ability decline in Aβ-induced AD rats by alleviating oxidative stress and inhibiting apoptosis-associated protein [27]. Fucoidan has a protective effect on the MPP+-induced PD cell model by improving mitochondrial dysfunction [28]. A study has shown that fucoidan could improve D-gal-induced cognitive impairment by increasing SOD and GSH levels, indicating that fucoidan had the potential to reduce oxidative stress [29]. Moreover, our group previously confirmed that fucoidan could relieve alcohol-induced depression-like behavior by reducing inflammation of the brain–gut axis [30]. However, whether fucoidan may improve mitochondrial dysfunction, attenuate inflammation and regulate gut microbiota in D-gal-exposed rats has not been reported yet.

In the current study, we aimed to assess the protective effect and possible mechanisms of fucoidan on cognitive dysfunction in D-gal-exposed rats.

## 2. Materials and Methods

### 2.1. Drugs and Reagents

According to previous descriptions, fucoidan was extracted from Saccharina japonica with an average MW of 251.2 kDa [31]. Briefly, the seaweed was chopped up and stirred overnight in water at a temperature of 70 °C. After centrifugation, the liquid mixture was mixed with calcium chloride. After centrifugation again, the supernatant of the solution was mixed with ethanol to make it fully react. Finally, the reaction products were collected and dried, which made up the sample of fucoidan. The content of sulfates was 28.0%, and the sample had a purity of 98.6%. D-gal (purity ≥99%) was derived from Sigma-Aldrich Chemical Co. (St Louis, MO, USA).

### 2.2. Animals

Healthy Sprague Dawley (SD) rats (8 weeks, SPF, 180 ± 20 g) were purchased from Beijing Vital River Laboratory Animal Technology Co., Ltd., Newstead, QLD, Australia. The rats were provided with standard rodent pellets food and tap water ad libitum, and kept in a temperature- and humidity-controlled room on a light–dark cycle (22 °C, 50–60% humidity, 12:12 h light–dark cycle). All rat experiments were approved by the Ethics Committee of Medical College of Qingdao University, and strictly adhered to the Guide for the Care and Use of Laboratory Animals of the National Institutes of Health (approval number: No. 20211008SD7520211217095).

### 2.3. Experimental Design

**Figure 1:**
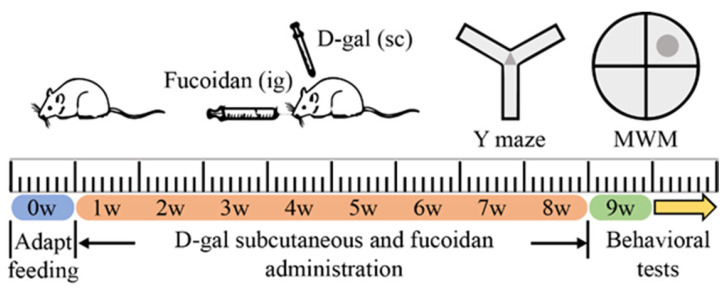
Experimental design flowchart.

After adaptive feeding for one week, SPF male SD rats were separated into five groups of fifteen in random order. Control group (CON): the rats were subjected to subcutaneous injection of distilled water, and then received a saline vehicle by oral gavage. Model group (MOD): the rats were injected daily subcutaneously with D-gal (200 mg/kg); then, the rats received a saline vehicle by oral gavage. Fucoidan control group (FCON): fucoidan (200 mg/kg) was administered by oral gavage to rats after they had been subcutaneously injected with distilled water. Low-dose fucoidan group (LF) and high-dose fucoidan group (HF): the rats of these two groups were injected daily subcutaneously with 200 mg/kg body weight D-gal, and received fucoidan (100 mg/kg and 200 mg/kg) by gavage. The treatments were given once a day for 8 consecutive weeks. All rats were weighed before the experiment, then once weekly over 8 weeks. After the 56th day of intervention, a behavioral test was carried out for 7 days. Anesthesia was administered to rats (5 per group) after the MWM test and 0.9% saline was transcardially infused into their hearts, and then perfused with 4% paraformaldehyde until the extremities appeared white. The brains were collected and stored in 4% paraformaldehyde for subsequent HE staining, Nissl staining, and immunofluorescence to analyze histopathological variations. In the other rats, blood and tissue (brains and hippocampus) were quickly collected after anesthesia, flash frozen, and stored immediately at −80 °C. Figure 1 indicates the flowchart of the experimental protocol.

### 2.4. Behavioral Test

#### 2.4.1. Y-Maze

Y-maze was performed as described previously [32]. The Y-maze is made of three gray polyethylene plastic arms with a 120° angle between every two arms, which are the familiar arm, start arm, novel arm and center area, respectively. First, we used a short board of the same color to block the entrance of novel arm; each rat started from the start arm, with the head facing the center area (the same for all rat) at the beginning of the test. Then, the rat could explore freely through the maze during a 5 min. Next, the rat was removed from the maze for an interval of 2 min, and the whole maze was wiped with 75% ethanol before removing the short board. The rat was advanced to the next phase when the short board was removed. The rat was allowed to maintain the same position and then freely explore the Y-maze for 5 min; meanwhile, the freely explorative movement and exercise time in the Y-maze were recorded using SMARTV3.0 software (Panlab Harvard Apparatus, Barcelona, Spain).

#### 2.4.2. Morris Water Maze

The Morris water maze (MWM) test was conducted according to previous description [33]. The water maze is a round pool and sectioned into four equal quadrants (I, II, III, IV). During the hidden platform training, the platform was kept in quadrant I (1 cm beneath the water surface). The rats were put into the water in any quadrant, and the time taken by each rat to reach the hidden platform was recorded (escape latency time). A training time of over 60 s was recorded as 60 s. The platform was removed to allow for probe testing on the sixth day. During the place navigation test, a target quadrant was identified for the platform’s placement. Then, the rats could explore the maze for 60 s. The frequency of crossing the target quadrant and the rats’ time of staying in the target quadrant were retained and calculated. The swimming path covered to locate the platform was tracked by a video tracking system (SMARTV3.0 software).

### 2.5. HE Staining and Nissl Staining

An optical microscope was used to observe pathological changes in the hippocampus area. In brief, the brain samples of the rats were sequentially dehydrated in a graded alcohol series (70%, 85%, 90%, 95%, and 100%). HE staining was performed on paraffin-embedded brain tissue sections after being dewaxed and rehydrated. The images were photographed by an optical microscope.

The neuronal damage in the hippocampus was evaluated using Nissl staining. Briefly, brain paraffin sections were immersed in toluidine blue solution for 10 min, differentiated in 95% ethanol for 10 min, and then sealed in neutral resin after treatment with xylene. The images were photographed by an optical microscope, and the living cell number was analyzed by Image J software (version 1.8.0).

### 2.6. Oxidative Stress and Inflammation Detection

DCFH-DA (Biosharp Co., Ltd., Beijing, China) and flow cytometry were used to detect changes in intracellular ROS levels. Briefly, fresh brain tissues were prepared in a single-cell suspension and then incubated with 10 μM H2DCFDA. The cells were washed and collected for fluorescence detection by flow cytometry.

CAT, SOD, GSH-Px, MDA, IL-1β, TNF-α, and IL-6 levels in the brain and Serum LPS levels in the serum were analyzed with the corresponding kits (Boster, Wuhan, China). The brain tissues were prepared as 10% tissue homogenate in a homogenizer and protein was quantified with a BCA kit (Dalian Meilun Biological Technology Co., Ltd., Dalian, China) [34].

### 2.7. ATP and mtDNA Copy Number Detection

A chemiluminescence assay kit (Nanjing Jiancheng Corp., Nanjing, China) was used to measure the ATP content. Briefly, after ATP working solution rested for 5 min, a 20 µL sample was added to a black 96-well plate. The ATP levels were estimated according to the ATP standard curve. Also, the mtDNA copy number in hippocampal tissues was analyzed by RT-PCR with SYBR Green kit (TaKaRa, Tokyo, Japan).

### 2.8. Immunofluorescence Staining

Immunofluorescence staining was carried out on paraffin-embedded brain tissue sections. Briefly, anti-DRP1 (1:100; Affinity Biosciences, OH, USA) or anti-SIRT1 antibodies (1:200; Servicebio, Wuhan, China) were incubated on the sections for 10 h, and the secondary antibody (1:300; Servicebio, Wuhan, China) was then incubated on the sections for 50 min. Lastly, the sections were counterstained with DAPI before observation by a fluorescence microscope. In this study, the mean fluorescence intensity of images was calculated by Image J software (version 1.8.0).

### 2.9. Western Blotting

The total protein was extracted by lysate. A BCA kit was used to quantify protein concentration; then, protein was transferred to the PVDF membrane, and after being resolved by SDS-PAGE (Millipore, Carrigtwohill, Ireland), DRP1 (1:500; Affinity Biosciences, OH, USA), MFN2 (1:1000; Affinity Biosciences, OH, USA), Adiponectin (APN, 1:1000; Zen BioScience, Chengdu, China), AMPK (1:1000; Affinity Biosciences, OH, USA), p-AMPK (1:1000; Affinity Biosciences, OH, USA), SIRT1 (1:2000; Boster, Wuhan, China), PGC-1α (1:1000; Zen BioScience, Chengdu, China), NRF1 (1:1000; Zen BioScience, Chengdu, China), TFAM (1:2000; Affinity Biosciences, Chicago, IL, USA) and β-actin (1:1000; internal control, Boster, Wuhan, China) were incubated together with the membrane in TBST overnight. A TBST wash and 2 h incubation with secondary antibodies followed. Lastly, protein signals were detected with an ECL kit (Boster, Wuhan, China).

### 2.10. 16S rRNA Gene Microbiome Sequencing Analysis

Following the behavioral experiments, the intestinal microbiome of CON, MOD and HF group rats (6 in each group) was analyzed by 16S rDNA gene sequencing technology. Sequencing methods followed previous studies [35]. Briefly, a Genomic DNA extraction kit was used to extract the DNA in the sample. Computer sequencing (Illumina novaseq 6000) and end-paired reads (2 × 250 bp) were obtained. Then, the length, distribution and quantity of clean reads were analyzed. Additionally, the LEfSe algorithm was employed to investigate differences between the groups in terms of intestinal flora abundance to determine significant differences.

### 2.11. Statistical Analysis

SPSS 19 (SPSS, Chicago, IL, USA), SigmaPlot 12.5 (SigmaPlot Software, Chicago, IL, USA) and R software (version 3.6.3) were used for data analysis and graphic presentations. The differences between the groups were compared using one-way analysis of variance. The results were expressed as means ± standard deviation (SD). The significance of gut microbiota analysis was analyzed using a nonparametric ranking analysis (Kruskal–Wallis test). Correlations between mitochondrial-dysfunction-related indices, oxidative-stress-related indices, inflammation factors and gut microbiota were assessed using Spearman’s correlation analysis. Differences with a *p* < 0.05 were considered statistically significant.

## 3. Results

### 3.1. Effects of Fucoidan on Impairment of Learning and Memory Abilities in D-Gal-Exposed Rats

The purpose of this paper was to demonstrate the beneficial effect of fucoidan on cognitive dysfunction in D-gal-exposed rats, and the experimental procedure is shown in Figure 1. Rats in each group grew well throughout the experimental period, and weight differences between the groups were not significant (Figure S1; *p* > 0.05).

**Figure 2:**
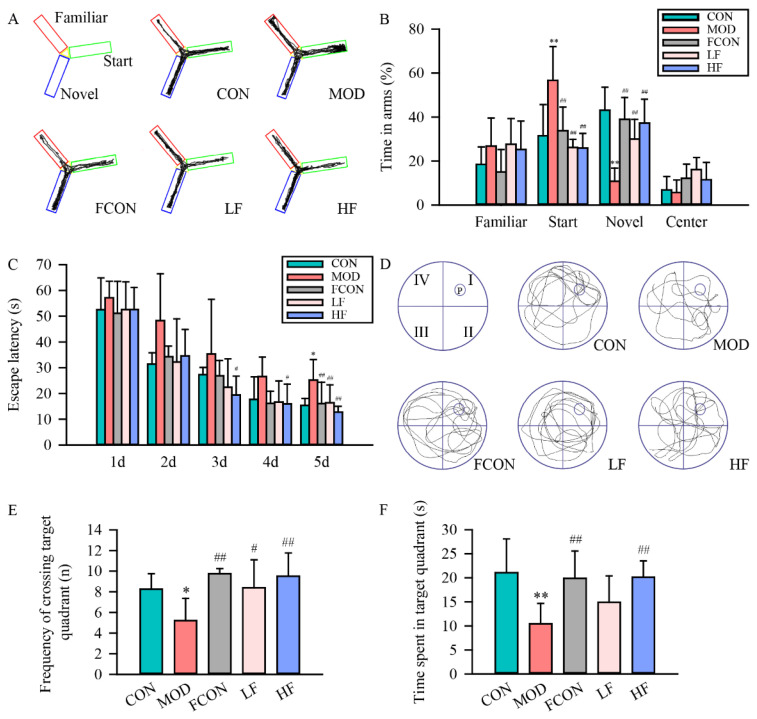
Fucoidan alleviated the learning and memory ability impairment induced by D-gal in rats. (**A**) Schematic representation and typical trajectories of rats in the Y-maze; (**B**) exploration time in each arm (Y-maze); (**C**) escape latency of each group within the training days; (**D**) representative movement traces of the MWM test on the 6th day; (**E**) indicated rats’ times of crossing the target quadrant; (**F**) probe percent time spent in target quadrant. Results are represented as mean ± SD (*n* = 15). * *p* < 0.05 and ** *p* < 0.01 vs. CON group, ^#^ *p* < 0.05 and ^##^ *p* < 0.01 vs. MOD group.

The Y-maze was applied to study the spatial recognition memory ability of animals, and the protective effect of fucoidan on spatial recognition memory dysfunction in D-gal-exposed rats was studied when the rats explored the start and novel arm. A schematic representation of the Y-maze and typical trajectories of rat in the Y-maze are shown in Figure 2A. The MOD group rats spent a significantly longer time in the start arm than those in the CON group (Figure 2B; *p* < 0.01), and a significantly shorter time exploring the novel arm than those in the CON group (Figure 2B; *p* < 0.01). In comparison with the MOD group, fucoidan intervention shortened the time in the start arm (Figure 2B; *p* < 0.01) and extended the time to explore the novel arm (Figure 2B; *p* < 0.01). A significant difference was not observed between the FCON and CON groups (Figure 2B; *p* > 0.05).

MWM was applied to reveal the spatial learning memory function. The results indicated that all groups had a gradual reduction in escape latency with the increase in training days (Figure 2C). As compared with the CON group, the MOD group’s escape latency significantly increased (Figure 2C; *p* < 0.05) in the last hidden platform test. At the same time, the fucoidan intervention group had a significantly shortened escape latency in comparison with the MOD group (Figure 2C; *p* < 0.01).

In the probe test, the typical trajectories of rats in the MWM, target quadrant crossing frequency, and target quadrant time are shown in Figure 2D–F. The results indicated that the MOD group had obvious cognitive deficits, and the target quadrant crossing frequency (Figure 2E; *p* < 0.05) and target quadrant time (Figure 2F; *p* < 0.01) of the MOD group significantly decreased compared with those of the CON group. The fucoidan intervention group had a significantly greater target quadrant crossing frequency (Figure 2E; LF: *p* < 0.05, HF: *p* < 0.01) and target quadrant time (Figure 2F; HF: *p* < 0.01) than the MOD group. A significant difference was not observed between the FCON and CON groups (*p* > 0.05).

### 3.2. Effects of Fucoidan on Histopathological Changes in Hippocampus Region in D-Gal-Exposed Rats

**Figure 3:**
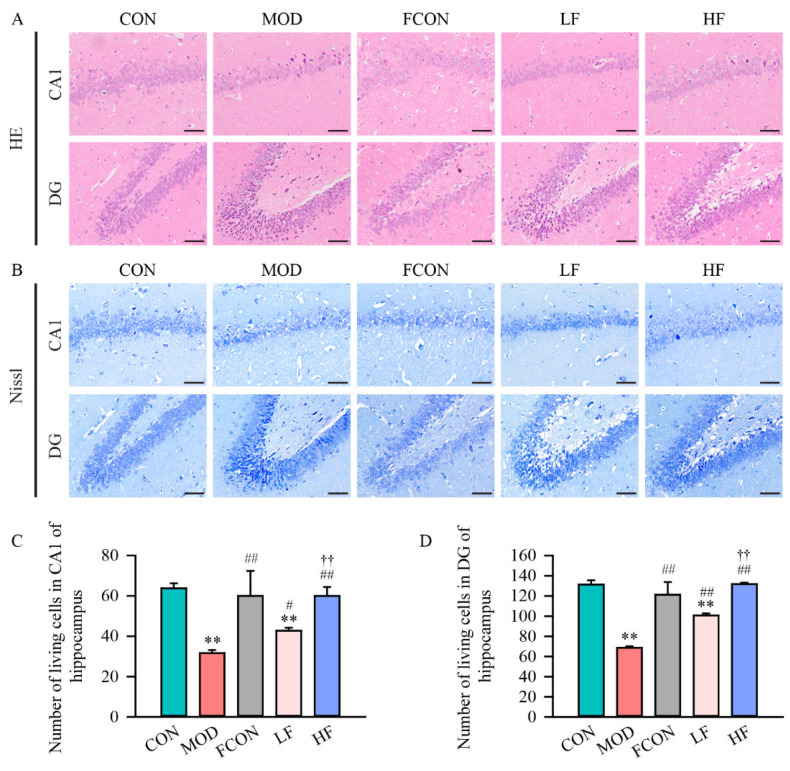
Fucoidan attenuated D-gal-induced histopathological changes in hippocampus region in rats. (**A**) The histopathological changes in each rat group’s hippocampal CA1 and DG regions were tested by HE staining; (**B**) the histopathological changes in hippocampal CA1 and DG regions were detected by Nissl staining; 20× magnification, the scale bar is 100 μm; number of living cells of (**C**) CA1 and (**D**) DG in hippocampus was analyzed. Results are shown as mean ± SD (*n* = 5). ** *p* < 0.01 vs. CON group, ^#^ *p* < 0.05 and ^##^ *p* < 0.01 vs. MOD group, ^††^ *p* < 0.01 vs. LF group.

The histopathological changes in the hippocampal CA1 and DG regions detected by HE staining are illustrated in Figure 3A. The CA1 pyramidal cells and the DG granule cells in the hippocampus of the CON and FCON groups had an explicit nuclear region and a visible nucleus without significant pathological changes. The hippocampal CA1 region and DG region cells in the MOD group showed a disorderly arrangement, nuclear pyknosis, and nuclear hyperchromasia. In comparison with the MOD group, the pathological changes in cells in CA1 and DG areas were attenuated after fucoidan intervention.

The results of Nissl staining in the hippocampus are presented in Figure 3B. As compared to the CON group, the cells in the hippocampal CA1 and DG area of the MOD group showed obvious damage, indistinct nuclear membrane, inconspicuous nucleolus, and nuclear hyperchromasia. The outcomes were scored as semi-quantitative to compare the histopathological changes in the hippocampal area of each group; the data demonstrated that the normal neuron number was significantly lower in the MOD group than in the CON group (CA1, *p* < 0.01, Figure 3C; DG, *p* < 0.01, Figure 3D). Interestingly, fucoidan alleviated neuronal injury and increased the number of normal neurons (CA1, LF: *p* < 0.05, HF: *p* < 0.01, Figure 3C; DG, *p* < 0.01, Figure 3D).

### 3.3. Effects of Fucoidan on Oxidative Stress and Inflammation in D-Gal-Exposed Rats

**Figure 4:**
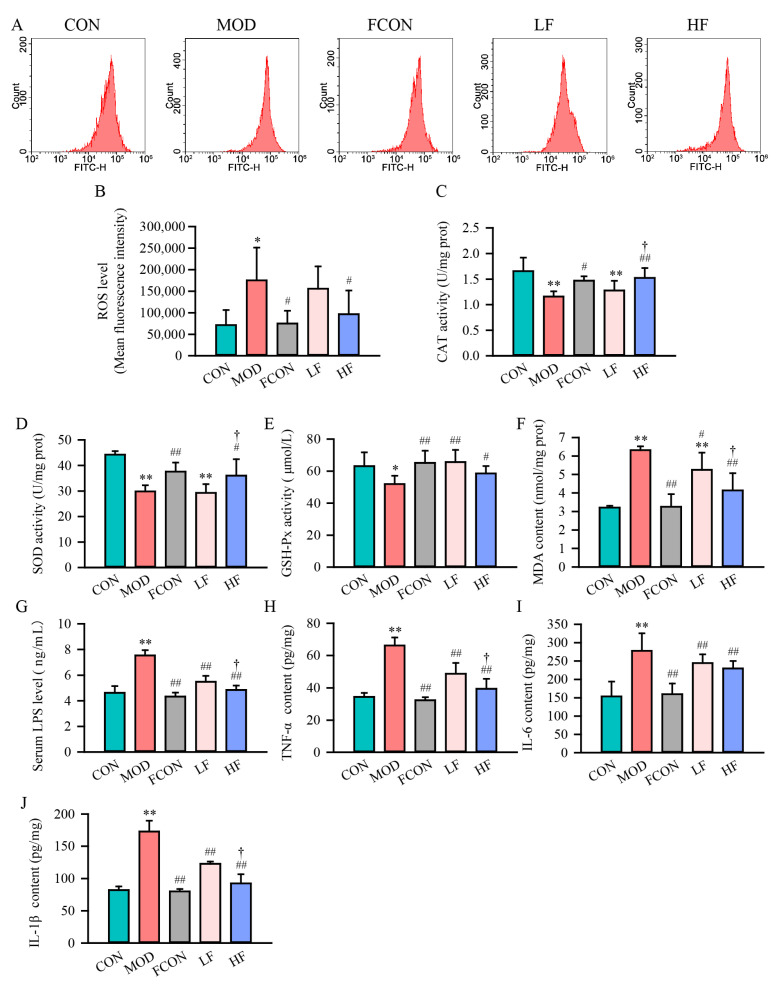
Fucoidan attenuated the oxidative stress and inflammation in D-gal-exposed rats. (**A**). Representative flow cytometry histograms of assays of ROS production and (**B**) mean fluorescence intensity of ROS probes in each group of cells; (**C**) CAT; (**D**) SOD; (**E**) GSH-Px; (**F**) MDA; (**H**) TNF-α; (**I**) IL-6 and (**J**) IL-1β content in brain tissue and (**G**) LPS level in each group of serum. Results are shown as mean ± SD (*n* = 5). * *p* < 0.05 and ** *p* < 0.01 vs. CON group, ^#^ *p* < 0.05 and ^##^ *p* < 0.01 vs. MOD group, ^†^ *p* < 0.05 vs. LF group.

Oxidative stress and inflammation have been demonstrated to play a significant part in aging [36,37]. To investigate whether fucoidan could reduce oxidative stress damage induced by D-gal, DCFH-DA probes were first used to detect ROS levels in each group (Figure 4A). The data demonstrated that the probe fluorescence’s intensity significantly increased in the MOD group in comparison to the CON group (Figure 4B; *p* < 0.05); the fluorescence intensity of the probe in the HF group was significantly reduced in comparison to that of the MOD group (Figure 4B; *p* < 0.05).

Furthermore, the activity of redox-related enzymes and malondialdehyde in rat brain tissue, the ultimate product of lipid oxidation, was measured using detection kits (Figure 4C–F). The data demonstrated that the CAT activity and SOD activity were significantly decreased in the MOD group in comparison with the CON group (*p* < 0.01). Also, the results showed that the CAT activity (Figure 4C; *p* < 0.01) and SOD activity (Figure 4D; *p* < 0.05) in the HF group were significantly higher than in the MOD group.

In comparison to the CON group, GSH-Px activity in the MOD group significantly reduced (Figure 4E; *p* < 0.05), while that in the LF group and the HF group significantly increased (Figure 4E; LF: *p* < 0.01, HF: *p* < 0.05). The MDA content in the MOD group significantly enhanced as compared with the CON group (Figure 4F; *p* < 0.01), while fucoidan reduced the content of MDA (Figure 4F; LF: *p* < 0.05, HF: *p* < 0.01). Moreover, in comparison to the LF group, the content of MDA significantly declined in the HF group (Figure 4F; *p* < 0.05).

No significant differences were observed in probe fluorescence’s intensity, CAT, GSH-Px, SOD and MDA levels of rat brain tissues in the FCON group as compared with the CON group (*p* > 0.05).

To probe whether fucoidan could reduce D-gal-induced inflammation, the levels of inflammation indicators (LPS, TNF-α, IL-6 and IL-1β) were detected in each group (Figure 4G–J). The results suggested that these indicators’ levels in the MOD group were significantly raised in contrast to the CON group (*p* < 0.01), while the levels significantly decreased after fucoidan intervention (*p* < 0.01) compared to the MOD group; also, the HF group had significantly declined LPS, TNF-α and IL-1β levels compared to the LF group (*p* < 0.05).

There were no significant differences in LPS, TNF-α, IL-6 and IL-1β levels in the FCON group in contrast to the CON group (*p* > 0.05).

### 3.4. Effects of Fucoidan on Mitochondrial Damage in D-Gal-Exposed Rats

**Figure 5:**
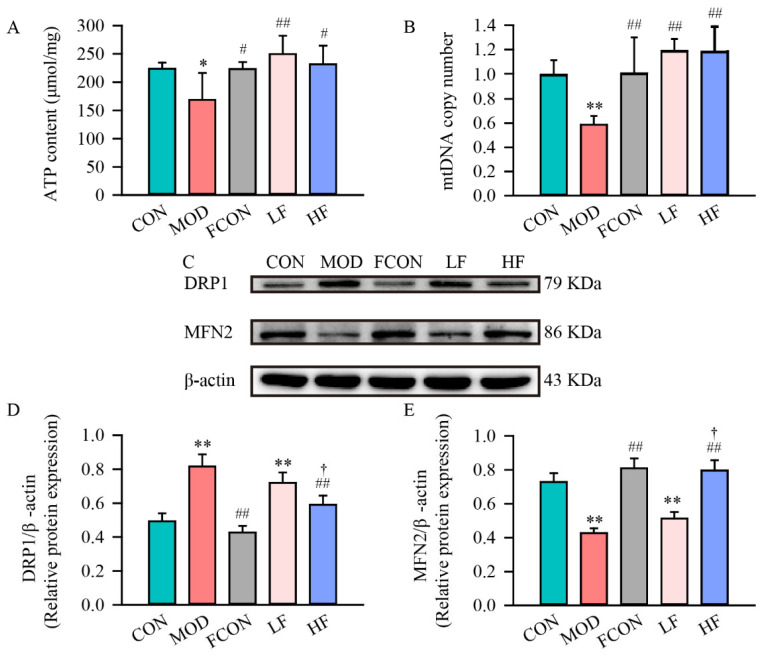
Fucoidan declined the mitochondrial damage of hippocampus of D-gal-exposed rats. (**A**) ATP content; (**B**) mtDNA copy number; (**C**) DRP1 expression and MFN2 expression were measured by Western blotting; semi-quantification of (**D**) DRP1 and (**E**) MFN2 protein expression. Data are represented as mean ± SD (*n* = 5). * *p* < 0.05 and ** *p* < 0.01 vs. CON group, ^#^ *p* < 0.05 and ^##^ *p* < 0.01 vs. MOD group, ^†^ *p* < 0.05 vs. LF group.

A major mechanism of brain aging, MCI, AD, and other diseases is mitochondrial dysfunction [38,39,40,41]. Mitochondria are also targets of ROS under oxidative stress, which leads to mitochondrial dysfunction [42]. To explore D-gal on mitochondrial function of rat hippocampus, we evaluated ATP content (Figure 5A) and mtDNA copy number (Figure 5B). The results indicated that compared with the CON group, ATP content in hippocampus significantly declined in the MOD group (Figure 5A; *p* < 0.05). Notably, hippocampus ATP content was restored to controls levels in the LF group and the HF group (Figure 5A; LF: *p* < 0.01, HF: *p* < 0.05).

Furthermore, in comparison with the CON group, the mtDNA copy number in the MOD group was significantly declined (Figure 5B; *p* < 0.01). As expected, after fucoidan intervention, the mtDNA copy number was restored to a similar level as that in the CON group (Figure 5B; *p* < 0.01).

In neurons, fission and fusion imbalances may result in mitochondrial structure and function impairment [43]. The DRP1 and MFN2 expression levels of the hippocampal tissue were measured by Western blotting (Figure 5C). The data suggested that the fission protein DRP1 expression level of the hippocampal tissue was significantly enhanced in the MOD group (Figure 5D; *p* < 0.01), and the fusion protein MFN2 expression level (Figure 5E; *p* < 0.01) was significantly declined in the MOD group in comparison with the CON group. Also, the DRP1 expression level in the HF group was significantly decreased (Figure 5D; *p* < 0.01), and the MFN2 expression level was significantly increased in comparison to the MOD group (Figure 5E; *p* < 0.01).

**Figure 6:**
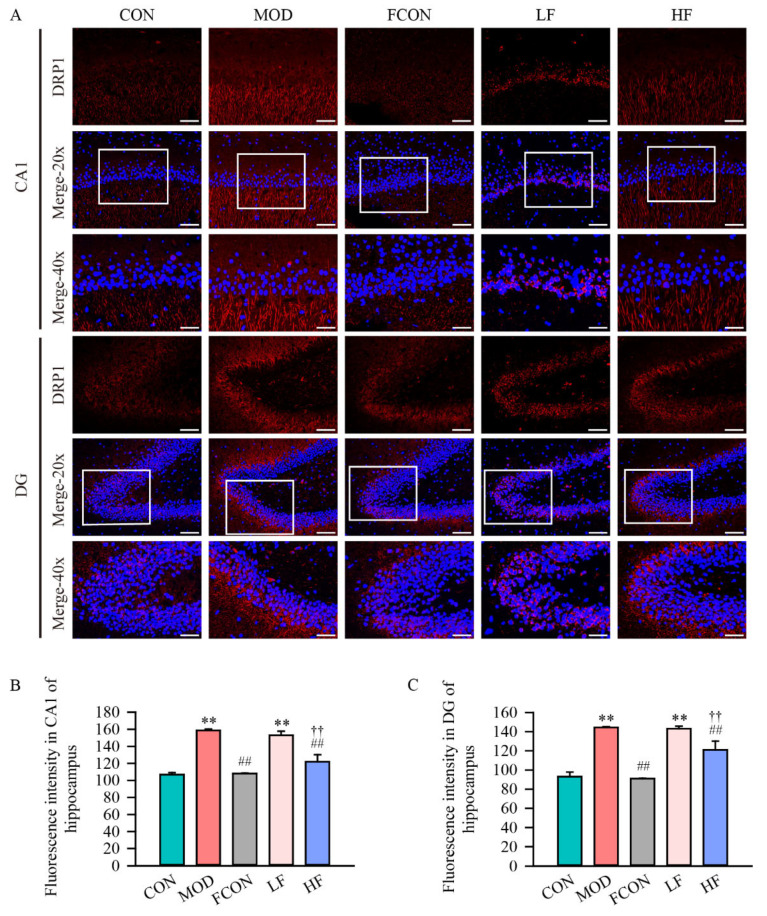
Fucoidan alleviated the fluorescent intensity of DRP1 in hippocampal area of D-gal-exposed rats. (**A**) Immunofluorescence images of DRP1 expression in red and nuclear staining in blue, white boxes region is magnified shown at 40× (DAPI, 20× magnification, the scale bar is 100 μm; 40× magnification, the scale bar is 50 μm); the fluorescent intensity of DRP1 in hippocampal area’s (**B**) CA1 region and (**C**) DG region was semi-quantified. Results are shown as mean ± SD (*n* = 4). ** *p* < 0.01 vs. CON group, ^##^ *p* < 0.01 vs. MOD group, ^††^ *p* < 0.01 vs. LF group.

Additionally, the effect of fucoidan on DRP1 protein expression in the hippocampal tissue was determined by immunofluorescence staining (Figure 6A). The data suggested that hippocampal tissue cells in the CA1 and DG regions contained DRP1 primarily in their cytoplasm. The fluorescence intensity for DRP1 in the hippocampal CA1 (Figure 6B; *p* < 0.01) and DG (Figure 6C; *p* < 0.01) areas was significantly enhanced in the MOD group in comparison to the CON group, whereas these areas showed decreased levels after treatment with high-dose fucoidan (CA1, Figure 6B, *p* < 0.01; DG, Figure 6C, *p* < 0.01).

### 3.5. Effects of Fucoidan on Mitochondrial Biogenesis of Hippocampal Tissue of D-Gal-Exposed Rats

**Figure 7:**
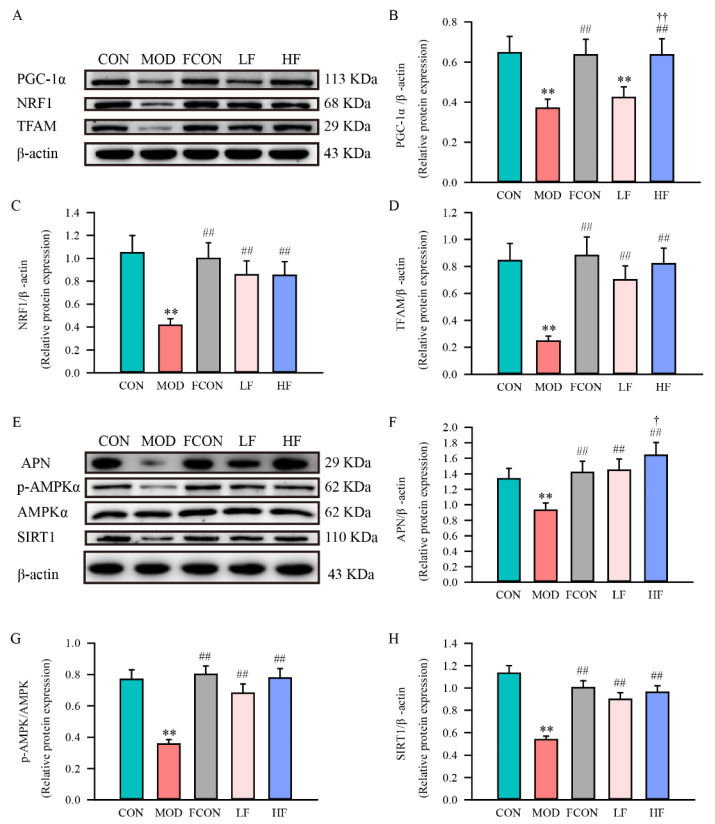
Fucoidan enhanced the mitochondrial biogenesis by regulating APN-AMPK-SIRT1 signaling pathway of hippocampal tissue in D-gal-exposed rats. (**A**) PGC-1α, NRF1 and TFAM expressions; semi-quantification of (**B**) PGC-1α, (**C**) NRF1 and (**D**) TFAM protein expression; (**E**) APN, p-AMPKα, AMPKα and SIRT1 expressions; semi-quantification of (**F**) APN, (**G**) p-AMPKα/AMPKα and (**H**) SIRT1 protein expression. Results are shown as mean ± SD (*n* = 3). ** *p* < 0.01 vs. CON group, ^##^ *p* < 0.01 vs. MOD group, ^†^ *p* < 0.05 and ^††^ *p* < 0.01 vs. LF group.

Mitochondrial biogenesis is the key mechanism to control mitochondrial renewal, and impaired mitochondrial biogenesis leads to mitochondrial dysfunction. To investigate whether mitochondrial dysfunction is associated with mitochondrial biogenesis, we examined the PGC-1α, NRF1 and TFAM expressions in the hippocampal tissue of each group (Figure 7A). The protein levels of PGC-1α (Figure 7B; *p* < 0.01), NRF1 (Figure 7C; *p* < 0.01) and TFAM (Figure 7D; *p* < 0.01) were significantly reduced in the MOD group in comparison to the CON group. However, fucoidan reversed the declined expressions of PGC-1α, NRF1 and TFAM in the hippocampal tissue of D-gal-exposed rats (*p* < 0.01).

A recent study found that APN-AMPK-SIRT1 can contribute to modulating mitochondrial biogenesis through upstream PGC-1α expression. Therefore, we investigated the APN, AMPKα, p-AMPKα (Thr172) and SIRT1 expressions of hippocampal tissue in each group (Figure 7E). The results demonstrated that the APN (Figure 7F; *p* < 0.01), p-AMPKα (Thr172)/AMPKα ratio (Figure 7G; *p* < 0.01) and SIRT1 (Figure 7H; *p* < 0.01) expressions of hippocampal were significantly decreased in the MOD group than in the CON group. Fucoidan intervention upregulated the SIRT1, APN and p-AMPKα (Thr172)/AMPKα ratio expressions (*p* < 0.01).

**Figure 8:**
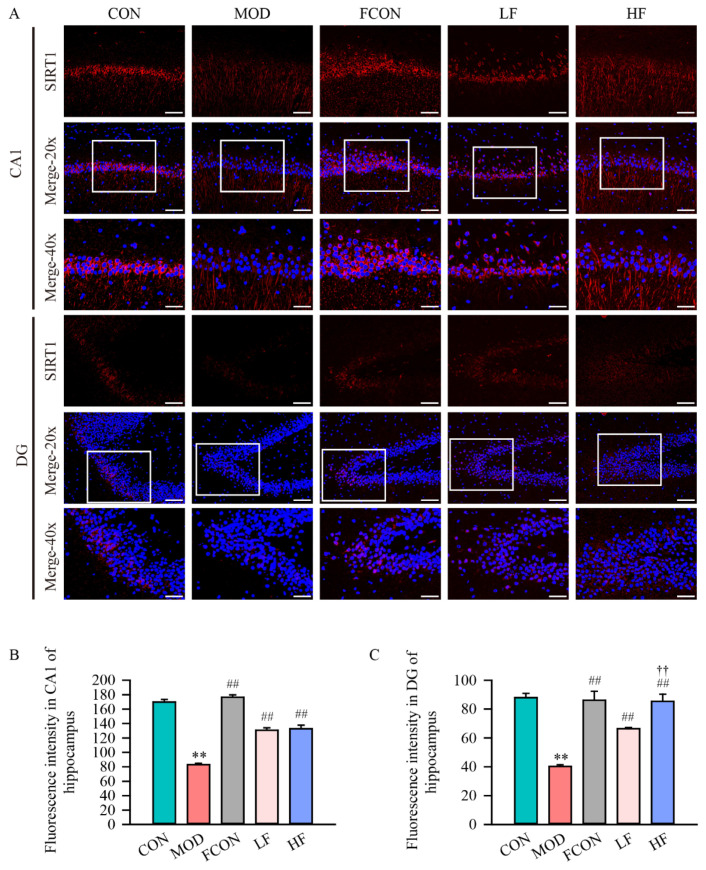
Fucoidan increased the fluorescent intensity of SIRT1 of hippocampus in D-gal-exposed rats. (**A**) Immunofluorescence images of SIRT1 expression in red and nuclear staining in blue, white boxes region is magnified shown at 40× (20× magnification, the scale bar is 100 μm; 40× magnification, the scale bar is 50 μm); the SIRT1 fluorescence intensity of hippocampal (**B**) CA1 region and (**C**) DG region was semi-quantified. Results are shown as mean ± SD (*n* = 4). ** *p* < 0.01 vs. CON group, ^##^ *p* < 0.01 vs. MOD group, ^††^ *p* < 0.01 vs. LF group.

Furthermore, the localization and expression intensity of SIRT1 in hippocampal tissue were detected by immunofluorescence (Figure 8A). The results demonstrated that SIRT1 expression could be detected both in the nucleus and cytoplasm of the CA1 and DG areas’ cells, and the SIRT1 fluorescent intensity in the hippocampal CA1 (Figure 8B; *p* < 0.01) and DG (Figure 8C; *p* < 0.01) areas significantly decreased in the MOD group in comparison to the CON group. But the SIRT1 fluorescent intensity was increased after fucoidan intervention (CA1, Figure 8B, *p* < 0.01; DG, Figure 8C, *p* < 0.01).

### 3.6. Effects of Fucoidan on Gut Microbiota in D-Gal-Exposed Rats

#### 3.6.1. α-Diversity Analysis

**Figure 9:**
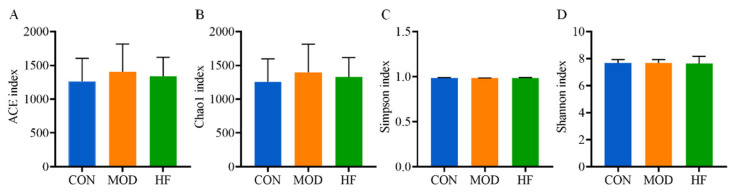
The α-diversity analysis of D-gal-exposed rats gut microbiota, including ACE (**A**), Chao1 (**B**), Simpson index (**C**), and Shannon index (**D**). Data are shown as mean ± SD (*n* = 6).

α-Diversity research data indicated that the ACE index (Figure 9A; *p* > 0.05) and Chao 1 index (Figure 9B; *p* > 0.05) slightly increased in the MOD group in comparison to the CON group, but no significant differences were found between the groups. Also, the results demonstrated that there existed no significant difference in intestinal flora species diversity among the groups according to the Simpson index (Figure 9C; *p* > 0.05) and Shannon index (Figure 9D; *p* > 0.05).

#### 3.6.2. β-Diversity Analysis

**Figure 10:**
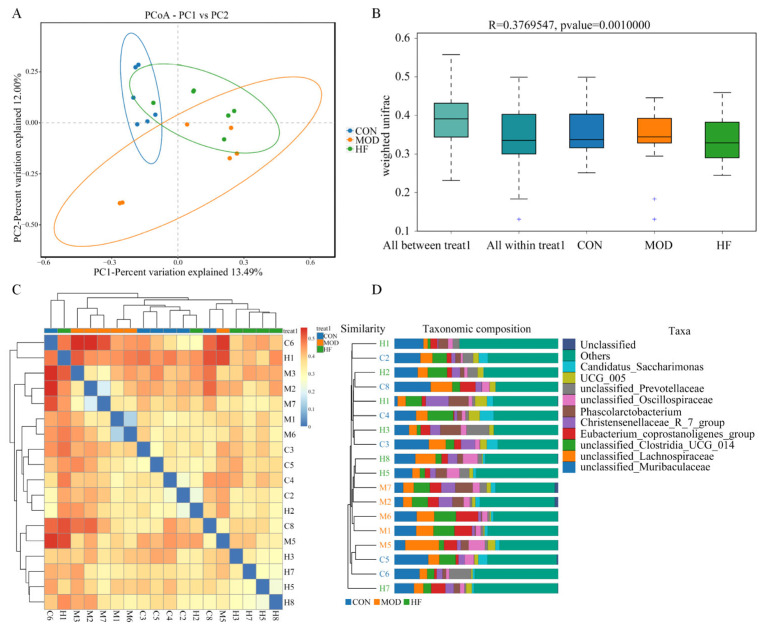
The β-diversity analysis of gut microbiota in D-gal-exposed rats. (**A**) Principal coordinate analysis between the three groups (PCoA); (**B**) ANOSIM assessed gut microbiota differences among three groups, + show outlier value; (**C**) β-diversity heatmap represented three groups gut microbiota variation and (**D**) UPGMA clustering of three groups microbiota.

As shown in Figure 10A, intestinal flora distribution was significantly different in the MOD group compared to the CON group, while the HF group was similar to the CON group, indicating that D-gal significantly altered intestinal microbiota composition and structure. Furthermore, the analysis of similarities’ (ANOSIM) results in Figure 10B showed that compared to the differences within groups, the differences between groups were more significant (R = 0.376, *p* = 0.001). The β-diversity heat mapping (Figure 10C) and UPGMA cluster mapping evaluation (Figure 10D) revealed that the composition of intestinal flora in the HF group has more similarity with that in the CON group than that between the CON group and the MOD group.

#### 3.6.3. Microbial Composition of the Phylum and Genus Level

**Figure 11:**
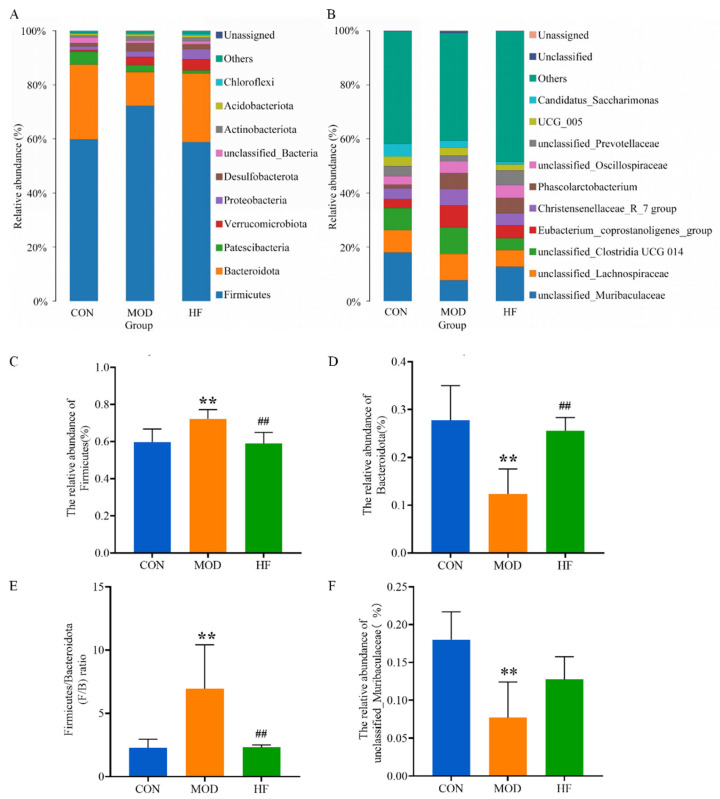
Gut microbiota community composition in each group. (**A**) phylum; (**B**) genus; (**C**) *Firmicutes* and (**D**) *Bacteroidota* abundance; (**E**) F/B ratios; (**F**) *unclassified_Muribaculaceae* abundance. ** *p* < 0.01 vs. CON group, ^##^ *p* < 0.01 vs. MOD group.

The main components of the intestinal microflora include *Bacteroidota*, *Firmicutes*, *Patescibacteria*, *Verrucomicrobiota* and *Proteobacteria* (Figure 11A) at the phylum level. Compared to the CON group, *Firmicutes’* abundance was significantly increased in the MOD group, while *Firmicutes’* abundance was restored after intervention with fucoidan (Figure 11C; *p* < 0.01). On the contrary, the abundance of *Bacteroidota* was significantly lower in the MOD group than in the CON group, and *Bacteroidota’s* abundance in the HF group significantly increased in comparison to the MOD group (Figure 11D; *p* < 0.01). The *Firmicutes*-to-*Bacteroidota* (F/B) ratios significantly enhanced in the MOD group compared to the CON group, while the F/B ratios significantly declined after fucoidan intervention (Figure 11E; *p* < 0.01). Figure 11B shows the ten bacteria genera with the highest abundance at the genus level. As compared to the CON group, *unclassified_muribaculaceae* abundance significantly decreased in the MOD group (Figure 11F; *p* < 0.01). Additionally, as compared with MOD (7.7% and 2.6%), *unclassified_muribaculaceae* and *Akkermansia* abundance was increased (12.8% and 3.7%) in the HF group, and *unclassified_Prevotellaceae* abundance (5.5%) was also higher in the HF group than in the MOD group (2%). No significant differences were observed in the abundance of *unclassified_muribaculaceae*, *Akkermansia* and *unclassified_Prevotellaceae* between the two groups (*p* > 0.05).

#### 3.6.4. LEfSe Analysis

**Figure 12:**
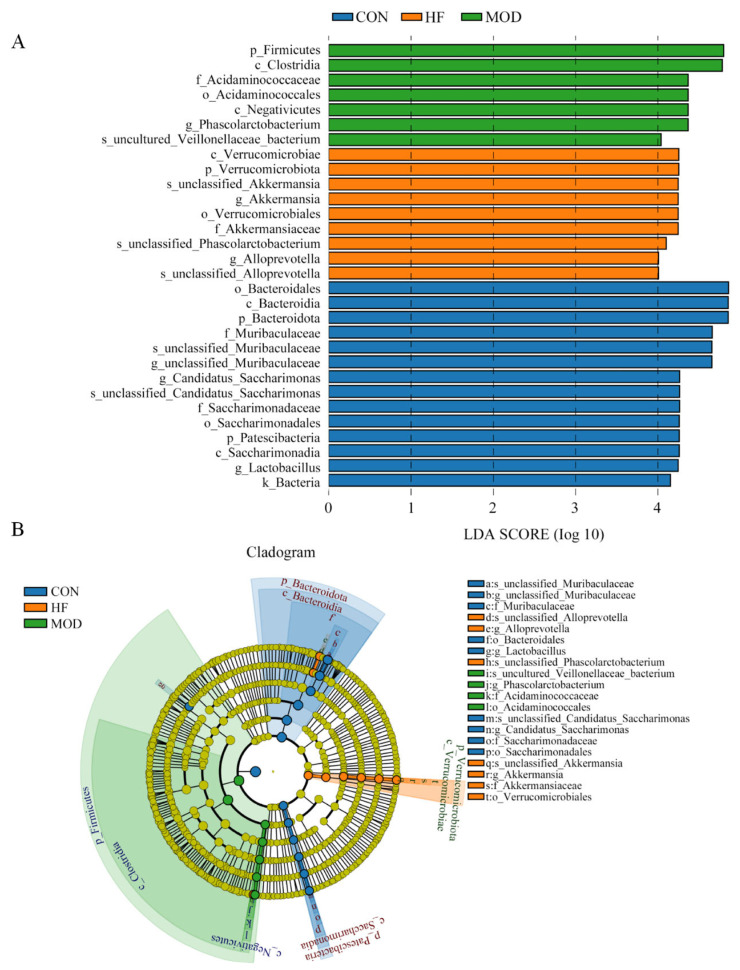
Cladogram for (**A**) LDA score and (**B**) LEfSe analysis. Only taxa with *p* < 0.05 and with an LDA score >4 are shown.

The LDA score and LEfSe results in Figure 12 show the specific bacteria between the three groups (LDA > 4). The major prevalent bacteria in the CON group were phylum *Bacteroidota* and genus *unclassified_Muribaculaceae*, phylum *Firmicutes* in the MOD group and genus *Akkermansia* in the HF group.

#### 3.6.5. Correlations among Mitochondrial-Dysfunction-Related Indices, Oxidative-Stress-Related Indices, Inflammation Factors and Gut Microbiota

**Figure 13:**
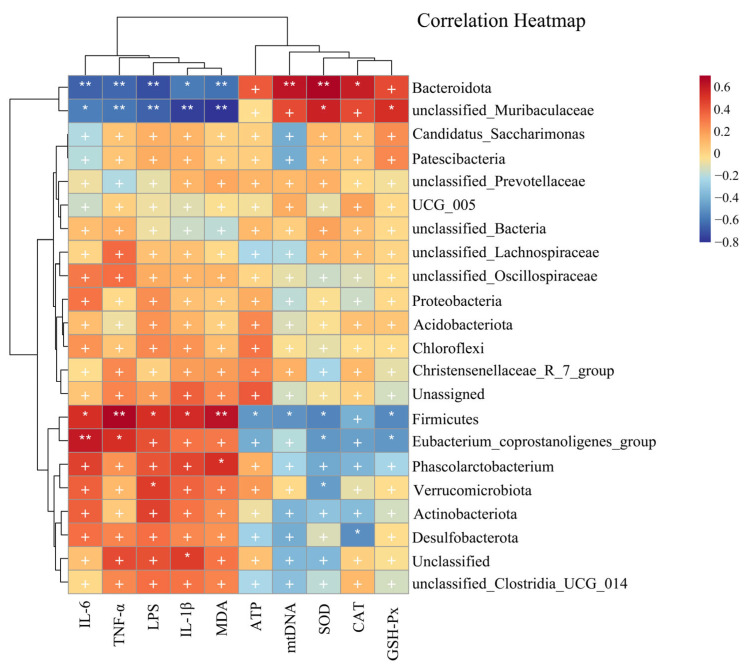
Correlation heatmap between biological index and gut microbiota. Correlations between biochemical parameters and gut microbiota are positively correlated in red, while negatively correlated in blue. Correlations are stronger with darker colors. * *p* < 0.05 and ** *p* < 0.01 show significant correlations, ^+^ *p* > 0.05 show no significant correlations.

The correlation between the ruminal fermentation parameters and bacteria at the genus and phylum levels was evaluated by performing Spearman’s correlation analysis as indicated in Figure 13. IL-6 and TNF-α levels demonstrated significantly positive correlation with the *Firmicutes* and *Eubacterium_copostanoligenes_group* (*p* < 0.05 or *p* < 0.01), while exhibiting a significantly negative correlation with *Bacteroidota* and *unclassified_Muribaculaceae* (*p* < 0.05 or *p* < 0.01). LPS exhibited a significantly positive correlation with *Firmicutes* and *Verrucomicrobiota* (*p* < 0.05), but correlated negatively with *Bacteroidota* and *unclassified_Muribaculaceae* (*p* < 0.01). IL-1β and MDA levels correlated positively with *Firmicutes* (*p* < 0.05), but displayed a significantly negative correlation with *Bacteroidota* and *unclassified_Muribaculaceae* (*p* < 0.05). ATP and mtDNA exhibited a significantly negative correlation with *Firmicutes* (*p* < 0.05), but mtDNA correlated positively with *Bacteroidota* (*p* < 0.01). SOD showed a significantly positive correlation with *Bacteroidota* and *unclassified_Muribaculaceae* (*p* < 0.05), but displayed a significantly negative correlation with *Firmicutes*, *Eubacterium_copostanoligenes_group* and *Verrucomicrobiota* (*p* < 0.05). The data suggested that CAT and *Bacteroidota* had a significant positive correlation (*p* < 0.05), while they correlated negatively with *Desulfobacterota* (*p* < 0.05). Finally, GSH-Px exhibited a significantly positive correlation with *unclassified_Muribaculaceae* (*p* < 0.05) and a significantly negative correlation with *Firmicutes* and *Eubacterium_copostanoligenes_group* (*p* < 0.05).

## 4. Discussion

D-gal exposure results in several age-like cognitive impairments, such as spatial recognition, memory, and learning damage. In the current study, we selected the Y-maze test and MWM to assess the impairment of cognitive ability in D-gal-exposed rats. The data of the Y-maze indicated that the time exploring the novelty arm was significantly decreased in D-gal-exposed rats. As regards the results of the MWM, the escape latency was significantly increased, the frequency of crossing the target quadrant was declined, and the time spent in the target quadrant was decreased in D-gal-exposed rats. To summarize, D-gal exposure could lead to the impairment of spatial recognition, memory, and learning abilities in rats, which was consistent with previous research [44,45]. The results suggested that fucoidan could alleviate the impairment of spatial learning and memory, and improve organizational behavior deficits in D-gal-exposed rats. This suggested that fucoidan had potential neuroprotective effects in restoring the impairment of cognitive and memory.

A sufficient number of neurons with synaptic connections are necessary for cognitive, learning, and memory functions [46]. In a previous study, D-gal exposure of rats resulted in disordered cell alignment, nuclear contraction, or apoptosis in the hippocampus [47]. Our data also showed that the normal cell number in the CA1 and DG regions of Nissl staining in hippocampal tissue of D-gal-exposed rats was reduced significantly, and the solid shrinkage cell number and disordered cell arrangement were increased. Fucoidan intervention improved cell morphological damage and significantly increased the cell number.

Elevated levels of oxidative stress are the main cause of induced loss of neurons and cognitive dysfunction. Overproduction of ROS and malfunctioning antioxidant defense mechanisms are symptoms of oxidative stress [48]. Our study suggested that fucoidan could significantly reduce ROS levels and MDA activity, and reverse the levels of CAT, SOD, and GSH-Px in D-gal-exposed rats, which is consistent with the results of previous research [29].

Next, we detected the ATP level and mtDNA copy number to evaluate the functional status in the mitochondria of hippocampal tissue. The results showed that mitochondrial ATP content in D-gal-exposed rats was significantly declined. It is worth noting that fucoidan had a preventive effect on ATP decline induced by D-gal, and ATP content returned to similarly normal levels in the LF and HF groups. Furthermore, the mtDNA copy number, a measure of quantifying mitochondrial damage, was significantly decreased in the hippocampus of D-gal-exposed rats, whereas fucoidan could substantially restore the mtDNA copy number. Studies have shown that fucoidan could significantly restore mitochondrial damage and exert a neuroprotective action by inhibiting the decrease in ATP content in a hydrogen peroxide-induced neurotoxicity model [49] and a trimethyltin-induced cognitive dysfunction model [50]. Moreover, fucoidan could up-regulate the mtDNA copy number in an ethanol-induced liver injury model [51]. This proves that fucoidan has the potential to regulate ATP content and the mtDNA copy number, which also confirms the above experimental results.

A lot of investigations have shown that mitochondrial dynamics and mitochondrial biogenesis play an essential part in the development of neurodegenerative diseases. Balance with mitochondrial dynamics is critical for mitochondrial health, with fusion involved in maintaining mitochondrial DNA integrity, mitochondrial apoptosis, and calcium signaling [52,53], and fission has been corroborated to play a key role in preventing mitochondrial oxidative injury and facilitating the degradation of damaged mitochondria [54]. A recent study has shown enhanced fission protein DRP1 and FIS1 expressions in AD patients, which strongly suggests the induction of mitochondrial fragmentation, while fusion protein MFN2, and OPA1 expression levels are reduced [55]. And in D-gal-exposed mouse models, the same situation occurred [56]. Our study is the first to suggest that fucoidan could significantly reduce the expression and fluorescence intensity of the fission protein DRP1, and increase fusion proteins’ MFN2 expression in D-gal-exposed rats.

It is mitochondrial biogenesis that controls mitochondrial turnover and number, and a reduction in mitochondrial biogenesis may result in dysfunctional mitochondria. PGC-1α, as a transcriptional coactivator, is important for mitochondrial biogenesis and mitochondrial function [57,58]. PGC-1α can interact with NRF1 and promote its expression, thereby increasing downstream TFAM expression and promoting mtDNA replication. It was recently shown that PGC-1α-NRF1-TFAM expression down-regulation was observed in AD patients [59]. In this study, we also found that PGC-1α-NRF1-TFAM expression was down-regulated in D-gal-exposed rats. However, it is unclear whether fucoidan can improve mitochondrial dysfunction and alleviate cognitive function in D-gal-exposed rats by increasing mitochondrial biogenesis. In the present research, we studied the beneficial effects of fucoidan on mitochondrial biogenesis in D-gal-exposed rats. Western blotting showed that fucoidan could significantly increase PGC-1α, NRF1 and TFAM expression levels. It follows that fucoidan could promote mitochondrial biogenesis by promoting the PGC-1α-NRF1-TFAM signaling pathway, which can help improve mitochondrial dysfunction.

Adiponectin (APN) is a hormone produced through fat cells that regulates metabolic processes, improves insulin sensitivity, and has a variety of effects in the nervous system [60]. A study suggested that most adiponectin effects are mediated by APN bound to adiponectin receptor 1 (AdipoR1) and adiponectin receptor 2 [61]. AdipoR1 is widely distributed in the rat hippocampus, cortex, and hypothalamus [62], and studies have shown that APN binds to AdipoR1 and phosphorylates AMPK for neuroprotective effects [63,64]. Moreover, fucoidan has been suggested to upregulate the level of APN and reduce hepatic steatosis in NAFLD through the AdipoR1 cascade [65,66].

AMP-activated protein kinase (AMPK) takes an essential role in energy metabolism in hippocampal tissue cells, but it also prevents brain cognition damage by regulating neuroinflammation, autophagy and apoptosis [67,68]. AMPKα, one of the AMPKs family members, and p-AMPKα (Thr172) can activate silent information transcriptional regulator 1 (SIRT1). There is evidence that SIRT1 is important in the prevention of many neurological disorders, and a possible mechanism is associated with oxidative stress, energy metabolism, mitochondrial function and autophagy [69,70,71]. Additionally, a lot of research has shown that SIRT1 can promote the activation of PGC-1α [72,73].

Next, we detected APN, AMPKα, p-AMPKα(Thr172) and SIRT1 expression by Western blotting, and analyzed the expression positions and fluorescence intensity of SIRT1 by immunofluorescence in the rat hippocampus. Our results showed that the p-AMPKα(Thr172)/AMPKα ratio, and APN and SIRT1 protein expression levels declined in D-gal-exposed rats. This study showed that the APN-AMPK-SIRT1 signaling pathway is related to the D-gal-induced damage modulus mechanism. After treatment with fucoidan, we noticed that the p-AMPKα(Thr172)/AMPKα ratio, and APN and SIRT1 expression levels significantly enhanced in the HF group. Further, the fluorescence results of SIRT1 agree with the protein expression results. In summary, our study suggests that fucoidan may regulate mitochondrial biogenesis PGC-1α-NRF1-TFAM by activating the APN-AMPK-SIRT1 pathway.

A dysregulated gut microbiota has been related to a wide range of diseases, such as diabetes, atherosclerosis, and cognitive impairment associated with aging [74]. A 16S sequencing study was conducted to determine the abundance of microflora in cecal contents of rats in each group. It was found that *Firmicutes’* abundance was significantly declined and *Bacteroidota’s* abundance was significantly increased in the cecum contents after intervention with fucoidan compared with the MOD group. Compared with *Firmicutes*, *Bacteroidota* encode more carbohydrate-degrading enzymes [75], and functional oligosaccharides can provide a better growth environment for *Bacteroidota* than *Firmicutes*, thus helping *Bacteroidota* make full use of carbohydrates as its substrate [76]. Therefore, the intervention of fucoidan inhibits *Firmicutes’* abundance and promotes *Bacteroidota’s* abundance. Previous studies have shown that *Firmicutes* are closely related to impaired intestinal barrier and LPS leakage [77]. A potential mechanism for fucoidan anti-inflammatory effects is the inhibition of *Firmicutes* and the upregulation of *Bacteroidota*. Genus *unclassified_Lachnospiraceae’s* abundance was decreased and the abundance of *unclassified_Muribaculaceae* was increased after intervention with fucoidan in comparison with the MOD group. *Muribaculaceae* has been suggested to regulate oxidative stress and inflammation via the microbiota–gut–brain axis, and to modulate cognitive function in AD patients [78,79]. In addition, *Akkermansia* was the dominant bacterial group in the HF group in LDA score. Studies have shown that ingestion of *Akkermansia* can fully improve intestinal diseases and extend the healthy life span in elderly mice. This may be related to the cognitive protective function of fucoidan [80]. According to the above results, it can be concluded that fucoidan can decline the level of inflammation and oxidative stress, alleviate mitochondrial dysfunction, and improve D-gal-induced cognitive dysfunction by regulating the structure and composition of rat microbial community.

## 5. Conclusions

**Figure 14:**
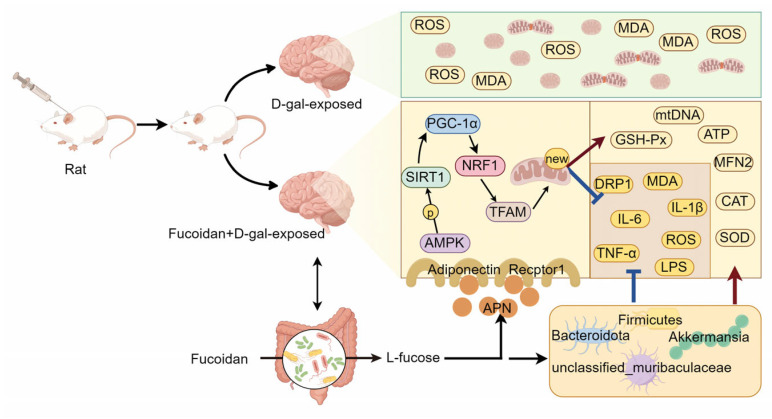
Potential alleviation mechanisms of fucoidan on cognitive impairment in D-gal-exposed rats.

In summary, our results showed that fucoidan can reduce oxidative stress and inflammation levels, and improve mitochondrial dysfunction to attenuate D-gal induced cognitive dysfunction by regulating APN-AMPK-SIRT1 signaling pathway and intestinal flora homeostasis (Figure 14). It is suggested that fucoidan may be a potential functional food for the prevention of age-related cognitive impairment. In order to carry out future work, further research on the mechanism of action of different tissues of rats is needed.

## Data Availability

The original contributions presented in the study are included in the article/Supplementary Materials, further inquiries can be directed to the corresponding author due to privacy reasons.

## Supplementary Materials

The following supporting information can be downloaded at: https://www.mdpi.com/article/10.3390/nu16101512/s1, Figure S1: Body weight among the groups during the experimental period.
