# Supplementary figures and images for: Fucoidan Improves D-Galactose-Induced Cognitive Dysfunction by Promoting Mitochondrial Biogenesis and Maintaining Gut Microbiome Homeostasis
Source: Nutrients. 2024 May 17;16(10):1512. doi: 10.3390/nu16101512 (PMC11124141; doi:10.3390/nu16101512)

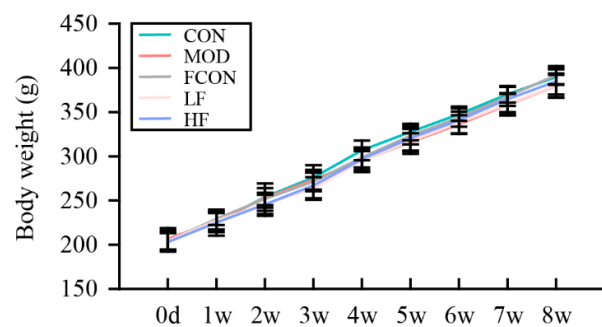

**Figure S1.** Body weight among the groups during the experimental period.

Supplement: Supplementary file 1 [file nutrients-16-01512-s001.zip › nutrients-2984654-supplementary.pdf]
